# The Efficacy of Combined Use of Huaier Granules in the Treatment of Primary Liver Cancer: An Updated Systematic Review and Meta-Analysis

**DOI:** 10.3390/ph18060884

**Published:** 2025-06-13

**Authors:** Tianhui Zhou, Yingying Zhang, Yuqing Lu, Zhaodong Sun, Dehui Wang, Xiaolong Zhang, Yu Wang, Yinuo Wei, Tiange Zhang, Xin Zhang, Ruohan Wei, Po Hu, Guangming Yang, Xinzhu Wang, Yang Pan

**Affiliations:** 1 School of Pharmacy, Nanjing University of Chinese Medicine, 138 Xianlin Avenue, Qixia District, Nanjing 210023, China; zhoutianhui36@163.com (T.Z.); yingyingzhan@student.unimelb.edu.au (Y.Z.); luyuqing2023@163.com (Y.L.); sunzhaodong1102@163.com (Z.S.); wdhqiao@foxmail.com (D.W.); zhangxiaolong4502@163.com (X.Z.); 042122109@njucm.edu.cn (Y.W.); 042122135@njucm.edu.cn (Y.W.); 15052201188@163.com (T.Z.); 042122110@njucm.edu.cn (X.Z.); 15365018755@163.com (R.W.); hupo_cpu@foxmail.com (P.H.); ygm@njucm.edu.cn (G.Y.); 2 Department of Surgery, The Royal Melbourne Hospital, The University of Melbourne, Parkville, VIC 3050, Australia; 3 Institute of Pharmaceutical Sciences, King’s College London, Franklin-Wilkins Building, 150 Stamford Street, London SE1 9NH, UK

**Keywords:** traditional Chinese medicine, Huaier granules, primary liver cancer, meta-analysis, drug combination, immune indices

## Abstract

**Objective:** Existing clinical data suggest that Huaier granules effectively improve the condition of PLC patients. However, their specific efficacy and safety in PLC patients remain unclear. This study aimed to investigate the effects of Huaier granules as an adjunctive therapy for PLC patients. **Methods:** This cohort study includes 4577 PLC patients, with data retrieved up to 2024. The patients were divided into the Huaier group (*n* = 2404) and a control group (*n* = 2173), and the treatment effects and safety between the two groups were compared. Review Manager 5.3 was used to analyze the clinical data. The fixed-effects and random-effects models were adopted. Stata 17.0 was used for sensitivity and bias analyses to evaluate publication bias. **Results:** Heterogeneity testing and analysis using a random-effects model showed that the combination of Huaier granules and conventional treatment significantly reduced the recurrence rate (OR = 0.65, 95% CI 0.50–0.85, *p* = 0.002) and improved the one-year survival rate (OR = 0.79, 95% CI 0.65–0.96, *p* = 0.02). Huaier granules also improved quality of life, reduced AFP levels (*p* < 0.00001), and significantly impacted immune function by altering the levels of the T lymphocyte subtypes CD^3+^, CD^4+^, CD^8+^ and the CD^4+^/CD^8+^ ratio. When Huaier granules were used in combination with other medications, no significant changes in side effects were observed. **Conclusions:** Huaier combination therapy shows good therapeutic efficacy and safety in PLC patients. However, this conclusion needs to be further validated through prospective clinical studies.

## 1. Introduction

Primary liver cancer (PLC) remains one of the most common cancers worldwide [1]. According to the latest report from the World Health Organization (WHO), there are over 900k new cases of liver cancer worldwide each year [2], with annual deaths ranging from 600k to 900k [3], and the incidence is expected to continue rising in the future. China, which ranks first globally in liver cancer incidence, reports approximately 360k new cases and 310k deaths annually [4]. In China, the incidence rate of liver cancer ranks fourth among all cancers, and the mortality rate ranks second, surpassed only by lung cancer [5,6]. It is noteworthy that PLC patients have a high recurrence rate and a five-year postoperative survival rate of less than 5% [7], indicating that PLC poses a significant threat to human health.

Concerning current PLC treatment options [8], the most commonly used targeted therapy is sorafenib [9], which treats cancer by inhibiting tumor angiogenesis and cell proliferation. This therapy can effectively extend patient survival; however, its efficacy is limited, it causes multiple side effects [10,11], and recurrence or metastasis occur following monotherapy [12]. Clinically, drug combinations have been employed to improve treatment outcomes. Recent studies showed that combining sorafenib with carboplatin and paclitaxel significantly improved efficacy and safety [13]. This suggests that combination therapy has promising potential for treating primary liver cancer, prompting numerous subsequent studies to focus on this approach.

In recent years, traditional Chinese medicine has gained significant attention as an adjuvant cancer therapy and has shown promising clinical outcomes. It demonstrates potential, particularly in enhancing treatment efficacy, reducing adverse effects, and improving patients’ quality of life. Huaier granules (trade name Jinke Huaier Keli), a traditional Chinese medicine preparation derived from the fruiting body of *Vanderbylia robiniophila* (Murrill), contain polysaccharide–protein (PS-T) as the main active ingredient. PS-T is a heteropolysaccharide composed of six monosaccharides combined with a protein consisting of 18 amino acids. Huaier granules possess anti-tumor, immunomodulatory, and anti-metastatic properties [14]. It is approved by the NMPA (National Medical Products Administration) as an adjuvant drug for clinical use in liver cancer treatment, aiming to boost the body’s anti-tumor capabilities and mitigate the toxic side effects of radiotherapy and chemotherapy. Numerous studies have demonstrated the efficacy of Huaier granules as an adjunct in treating primary liver cancer. However, these studies are often scattered and lack consistency. Meta-analysis, as a systematic review method, allows for the comprehensive statistical analysis of multiple independent studies, addressing discrepancies between studies and enhancing statistical power. Although the therapeutic significance of Huaier granules in primary liver cancer has been recognized, previous studies have reported related meta-analyses of Huaier granules in treating various cancers [15,16]. Due to the current clinical approval of Huaier granules by the NMPA (National Medical Products Administration) as an adjuvant drug for liver cancer, a comprehensive and systematic evaluation of medication for liver cancer patients remains both necessary and meaningful. Based on previous research, we have comprehensively summarized the current state of Huaier combined therapy for liver cancer patients. The data are relatively complete, and we have discussed several advantages of Huaier combined therapy for liver cancer from a new perspective. This study conducts a meta-analysis of published research to summarize the therapeutic outcomes of Huaier granules in treating primary liver cancer, and it provides a theoretical foundation for the development of natural products from traditional Chinese medicine for primary liver cancer and assembles the scientific evidence for Huaier preparation to better serve the clinical treatment of primary liver cancer.

## 2. Materials and Methods

### 2.1. Literature Search Strategy

Literature research. A comprehensive systematic search of PubMed, Web of Science, and China National Knowledge Infrastructure (CNKI) was conducted from inception to 2024. The search strategy was as follows:

“huaier” [Supplementary Concept] OR “huaier” [All Fields] OR ((“primaries” [All Fields] OR “primary” [All Fields]) AND (“carcinoma, hepatocellular” [MeSH Terms] OR (“carcinoma” [All Fields] AND “hepatocellular” [All Fields]) OR “hepatocellular carcinoma” [All Fields] OR (“hepatic” [All Fields] AND “carcinoma” [All Fields]) OR “hepatic carcinoma” [All Fields])) OR ((“primaries” [All Fields] OR “primary” [All Fields]) AND (“liver neoplasms” [MeSH Terms] OR (“liver” [All Fields] AND “neoplasms” [All Fields]) OR “liver neoplasms” [All Fields] OR (“liver” [All Fields] AND “cancer” [All Fields]) OR “liver cancer” [All Fields])) OR (“clinical trial” [Publication Type] OR “clinical trials as topic” [MeSH Terms] OR “clinical trial” [All Fields]) OR (“j pathol clin res” [Journal] OR “clin res” [Journal] OR (“clinical” [All Fields] AND “research” [All Fields]) OR “clinical research” [All Fields]).

### 2.2. Inclusion and Exclusion Criteria

#### 2.2.1. Inclusion Criteria

1. Participants: patients with a definite diagnosis of primary liver cancer. 2. Intervention: surgical resection or treatment with Transcatheter Arterial Chemoembolization (TACE) or other drugs. 3. Comparison: whether Huaier granules were used or not. 4. Study design: a prospective study or a clinical randomized controlled trial. 5. Outcomes: the primary outcome was the recurrence rate; secondary outcomes included immune indicators (the T lymphocyte subtypes CD^3+^, CD^4+^, CD^8+^, and CD^4+^/CD^8+^), the alpha-fetoprotein change rate, and adverse reactions.

#### 2.2.2. Exclusion Criteria

1. Duplicate publication. 2. No clinical data were available. 3. The use of Huaier was not used as a control group.

### 2.3. Literature Screening and Data Extraction

The literature was independently screened by two reviewers (Tianhui Zhou and Xin Zhang). The basic information, the design of the observation control groups, and the outcome data were sorted out and extracted by Tianhui Zhou and Xin Zhang. Any inconsistencies were carefully reviewed by a third reviewer (Yu Wang).

### 2.4. Risk of Bias Assessment of the Included Studies

Two reviewers (Tianhui Zhou and Yu Wang) used the risk of bias assessment tool recommended by the Cochrane Handbook to evaluate the 35 selected studies in seven aspects: random sequence generation, allocation concealment, blinding of participants and staff, blinding of outcome assessment, incomplete outcome data, selective reporting, and other biases. Three categories of “high”, “low”, and “uncertain” were used to make judgments. Any inconsistencies were carefully evaluated by a third reviewer (Yingying Zhang).

### 2.5. Statistical Analysis

Review Manager 5.3. was used to analyze the clinical data. The count data of the control group and the experimental group were evaluated by the relative risk (RR) and 95% confidence interval (CI). The measurement data were analyzed by using the standardized mean difference (SMD) and 95% CI [17]. For the single-arm study without a control group, the event rate was analyzed by using the risk difference (RD, %) and 95% CI [18]. If I^2^ < 50% or *p* > 0.05, the heterogeneity among the included studies was considered small, and the fixed-effect model was adopted; otherwise, the random-effects model was adopted [19]. Therefore, the subsequent 7 meta-analyses selected models according to this standard. Stata 17.0 was used for sensitivity and bias analyses to assess publication bias.

### 2.6. Sensitivity and Subgroup Analyses

Sensitivity and subgroup analyses helped identify any inconsistencies or potential biases and provided a deeper understanding of the effects of Huaier combination therapy under different conditions. The sensitivity analysis was performed to assess the robustness of the findings by 1. excluding studies with a high risk of bias; 2. analyzing the impact of removing one study at a time on the overall results; and 3. evaluating the effect of small sample sizes on the overall conclusions. Subgroup analysis was conducted to explore the impact of specific variables on the outcomes. Subgroups included the total recurrence rate, one-year survival rate, quality-of-life analysis, T lymphocyte subtypes CD^3+^, CD^4+^, CD^8+^, CD^4+^/CD^8+^, AFP (alpha-fetoprotein), and side effects. These findings helped to clarify which factors had the most impact on the effectiveness of the treatments (see Table S1).

### 2.7. Compliance Statement

This systematic review was conducted and reported in accordance with the PRISMA guidelines, registration number CRD420251063132 (PROSPERO) (see Supplementary File S1, available online at https://www.prisma-statement.org/s/PRISMA_2020_checklist-fxke.docx (accessed on 26 February 2025)).

## 3. Results

### 3.1. Literature Retrieval Process and Results

**Figure 1:**
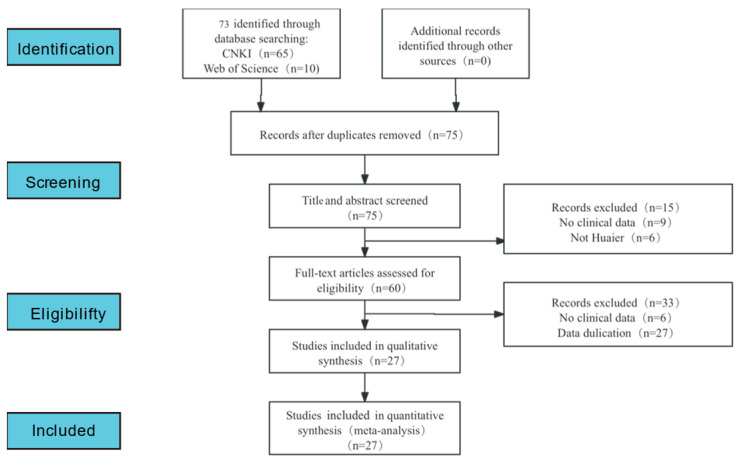
Flow chart for the study selection process.

A preliminary literature search was carried out on a total of 27 RCT studies (in alphabetical order by author’s initials (J.Y. Lei et al., 2015 [20]; Guangsheng Zhao et al., 2017 [21]; Lin Zhou et al., 2018 [22]; Zhen Wang et al., 2021 [23]; Shaoju Luo et al., 2023 [24]; Qian Chen et al., 2018 [25]; Jie Li et al., 2022 [26]; Xiangdong Hua et al., 2016 [27]; Jianjun Zhao et al., 2006 [28]; Wuhan Zhou et al., 2023 [29]; Hairui Wang et al., 2020 [30]; Hui Wu et al., 2014 [31]; Lin Zhang et al., 2023 [32]; Xingang Li et al., 2009 [33]; Gongliang Shi et al., 2024 [34]; Rui jiang et al., 2017 [35]; Yannan Kang et al., 2019 [36]; Liang Wang et al., 2021 [37]; Guanlei Wang et al., 2018 [38]; Changjie Wang et al., 2022 [39]; Shiguo Zhang et al., 2024 [40]; Lei Li et al., 2023 [41]; Jiannan Wu et al., 2022 [42]; Yuanren Gao et al., 2020 [43]; Yifei Tang et al., 2018 [44]; Dazhi Han et al., 2022 [45]; Xingpeng Shi et al., 2024 [46]) that qualified and were subsequently found. The search procedures are displayed in Figure 1.

### 3.2. Study Characteristics and Assessment of Risk of Bias

All studies were published before 2025 and sourced from authoritative hospitals in China. This meta-analysis involved 4577 participants: 2404 were designated as the experimental group, and 2173 were assigned to the control group. The basic characteristics are summarized in Table 1.

**Figure 2:**
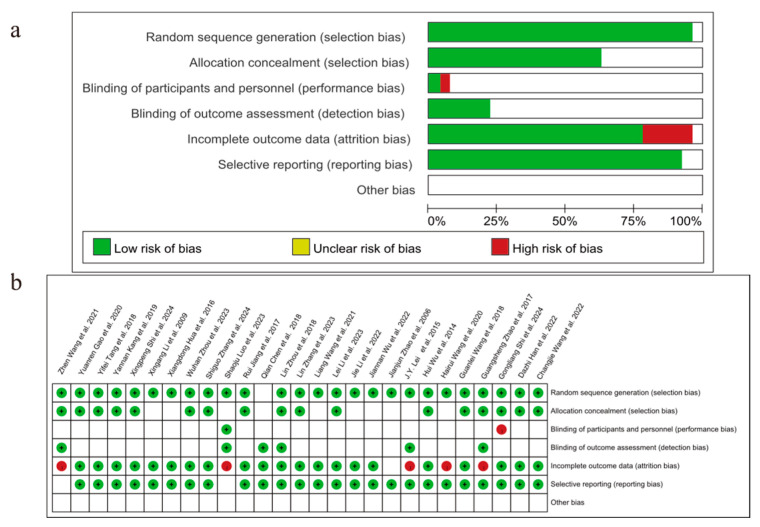
Included studies’ risk of bias plot: (**a**) summary, (**b**) traffic light ("+" indicated low risk (green) with high credibility of the results; "−" indicated high risk (red) with possible serious bias in the results). J.Y. Lei et al., 2015 [20]; Guangsheng Zhao et al., 2017 [21]; Lin Zhou et al., 2018 [22]; Zhen Wang et al., 2021 [23]; Shaoju Luo et al., 2023 [24]; Qian Chen et al., 2018 [25]; Jie Li et al., 2022 [26]; Xiangdong Hua et al., 2016 [27]; Jianjun Zhao et al., 2006 [28]; Wuhan Zhou et al., 2023 [29]; Hairui Wang et al., 2020 [30]; Hui Wu et al., 2014 [31]; Lin Zhang et al., 2023 [32]; Xingang Li et al., 2009 [33]; Gongliang Shi et al., 2024 [34]; Rui jiang et al., 2017 [35]; Yannan Kang et al., 2019 [36]; Liang Wang et al., 2021 [37]; Guanlei Wang et al., 2018 [38]; Changjie Wang et al., 2022 [39]; Shiguo Zhang et al., 2024 [40]; Lei Li et al., 2023 [41]; Jiannan Wu et al., 2022 [42]; Yuanren Gao et al., 2020 [43]; Yifei Tang et al., 2018 [44]; Dazhi Han et al., 2022 [45]; Xingpeng Shi et al., 2024 [46].

The assessment of risk biases is outlined in Figure 2 ((a) summary, (b) traffic light). In general, the overall methodological qualities of the included studies were poor to moderate.

### 3.3. Meta-Analysis Results

#### 3.3.1. Meta-Analysis of Total Recurrence Rate

**Figure 3:**
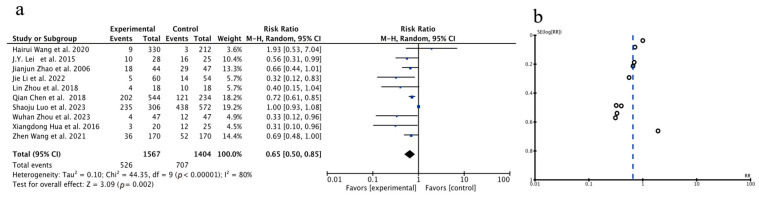
Forest plot (**a**) and funnel plot (**b**) of total recurrence rate. J.Y. Lei et al., 2015 [20]; Lin Zhou et al., 2018 [22]; Zhen Wang et al., 2021 [23]; Shaoju Luo et al., 2023 [24]; Qian Chen et al., 2018 [25]; Jie Li et al., 2022 [26]; Xiangdong Hua et al., 2016 [27]; Jianjun Zhao et al., 2006 [28]; Wuhan Zhou et al., 2023 [29]; Hairui Wang et al., 2020 [30].

A total of 10 studies were included in the analysis of the overall recurrence rate. Given the results of the heterogeneity test (*p* < 0.00001, I^2^ = 80%), a random-effects model was employed. Compared to the control group, the combination of Huaier treatment led to a significant reduction in the recurrence rate among patients [OR = 0.65, 95% CI (0.50, 0.85), *p* = 0.002], indicating that the recurrence rate in PLC patients was significantly lowered by treating with Huaier. A funnel plot was constructed, as shown in Figure 3 (Forest plot (a) and funnel plot (b)). Additionally, Begg’s test and Egger’s test were performed to assess publication bias using Stata 17. The *p*-values for Begg’s test and Egger’s test indicate that there is no evidence of publication bias in this study.

#### 3.3.2. Meta-Analysis of One-Year Survival Rate

**Figure 4:**
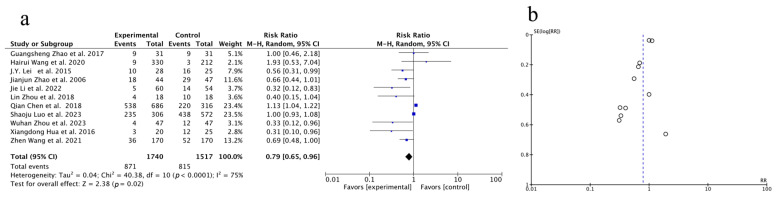
Forest plot (**a**) and funnel plot (**b**) of one-year survival rate. J.Y. Lei et al., 2015 [20]; Guangsheng Zhao et al., 2017 [21]; Lin Zhou et al., 2018 [22]; Zhen Wang et al., 2021 [23]; Shaoju Luo et al., 2023 [24]; Qian Chen et al., 2018 [25]; Jie Li et al., 2022 [26]; Xiangdong Hua et al., 2016 [27]; Jianjun Zhao et al., 2006 [28]; Wuhan Zhou et al., 2023 [29]; Hairui Wang et al., 2020 [30].

Preliminary data from 11 studies disclosed the total effective rate. Based on the results of the heterogeneity test (*p* < 0.0001, I^2^ = 75%), a random-effects model was applied for the analysis. The findings revealed a statistically significant increase in the total effective rate in the experimental group compared to the control group [OR = 0.79, 95% CI (0.65, 0.96), *p* = 0.02], indicating that the combination of Huaier therapy shows superior efficacy in treating PLC compared to treatment without Huaier. As the number of studies exceeded 10, a funnel plot was generated as illustrated in Figure 4 (Forest plot (a) and funnel plot (b)), and Begg’s test and Egger’s test were conducted using Stata 17 to quantify the publication bias. The *p*-values for both tests indicate that there is no evidence of publication bias in this study.

#### 3.3.3. Meta-Analysis of Quality-of-Life Analysis

**Figure 5:**
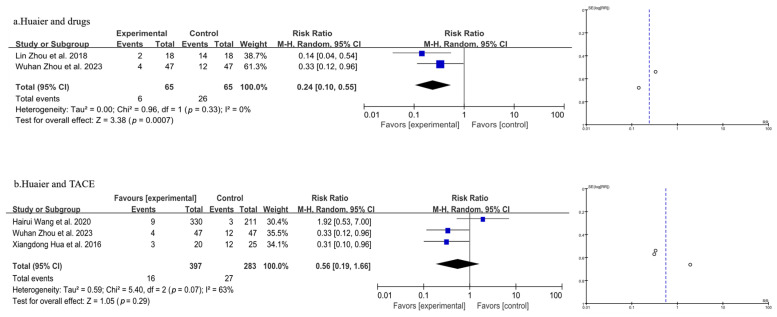
Forest plot and funnel plot of quality-of-life analysis (the combination of Huaier and drugs in (**a**) and the combination of Huaier and TACE in (**b**)). Lin Zhou et al., 2018 [22]; Xiangdong Hua et al., 2016 [27]; Wuhan Zhou et al., 2023 [29]; Hairui Wang et al., 2020 [30].

Five studies, involving a total of 810 patients, were included in the analysis, as shown in Figure 5 (the combination of Huaier and drugs in 5a and the combination of Huaier and TACE in 5b). The heterogeneity test results suggested a model using random effects (*p* = 0.33, I^2^ = 0% in 5a, and *p* = 0.07, I^2^ = 63% in 5b). Overall, the meta-analysis demonstrated that compared with the group without the use of Huaier adjuvant therapy, the drug group using Huaier adjuvant therapy and TACE reflected a different trend. In the drug group using Huaier adjuvant therapy, there was significant improvement in the quality of life of patients after treatment [OR = 0.24, 95% CI (0.10, 0.55), *p* = 0.0007] (Figure 5a). However, the combination of Huaier and TACE did not result in a significant change in the quality of life compared to TACE alone [*p* = 0.29, OR = 0.56, 95% CI (0.19, 1.66)] (Figure 5b).

#### 3.3.4. Meta-Analysis of T Lymphocyte Subtype CD^3+^

**Figure 6:**
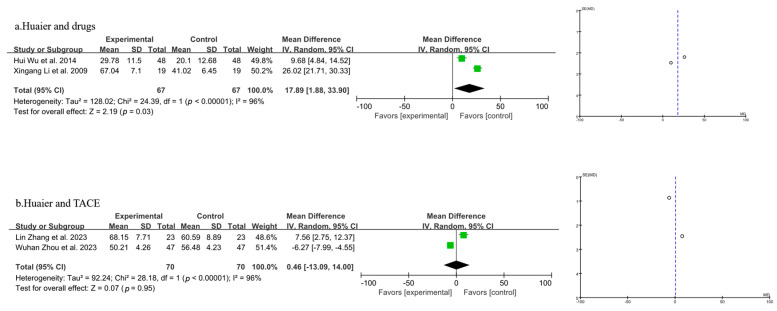
Forest plot and funnel plot of T lymphocyte subtype CD^3+^ levels (the combination of Huaier and drugs in (**a**) and the combination of Huaier and TACE in (**b**)). Wuhan Zhou et al., 2023 [29]; Hui Wu et al., 2014 [31]; Lin Zhang et al., 2023 [32]; Xingang Li et al., 2009 [33].

Four studies were included in the analysis of CD^3+^ levels, as shown in Figure 6 (the combination of Huaier and drugs in 6a and the combination of Huaier and TACE in 6b). A random-effects model was employed after the heterogeneity test indicated significant variability (*p* < 0.00001, I^2^ = 96%). The results indicated that the combination of Huaier and drug treatment for PLC led to a statistically significant decrease in CD^3+^ levels compared to drugs alone [MD = 17.89, 95% CI (1.88, 33.90), *p* = 0.03] (Figure 6a). Compared to the control group, the combination of Huaier and TACE did not result in a significant change in CD^3+^ levels among PLC patients [MD = 0.46, 95% CI (−13.09, 14.00), *p* = 0.95] (Figure 6b).

#### 3.3.5. Meta-Analysis of T Lymphocyte Subtype CD^4+^

**Figure 7:**
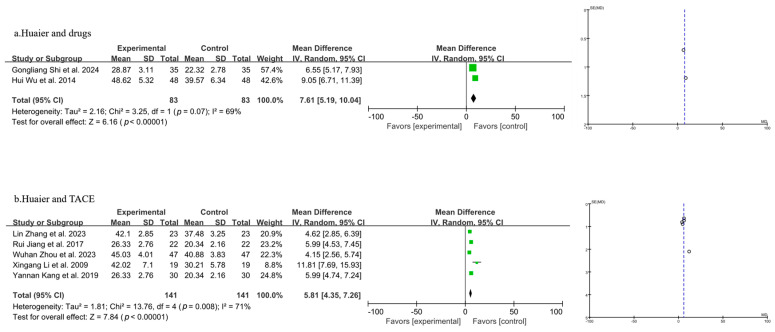
Forest plot and funnel plot of T lymphocyte subtype CD^4+^ levels (the combination of Huaier and drugs in (**a**) and the combination of Huaier and TACE in (**b**)). Wuhan Zhou et al., 2023 [29]; Hui Wu et al., 2014 [31]; Lin Zhang et al., 2023 [32]; Xingang Li et al., 2009 [33]; Gongliang Shi et al., 2024 [34]; Rui jiang et al., 2017 [35]; Yannan Kang et al., 2019 [36].

A total of seven studies were included in the analysis of CD^4+^ levels, as shown in Figure 7 (the combination of Huaier and drugs in 7a and the combination of Huaier and TACE in 7b). A random-effects model was applied following the heterogeneity test results. The study’s results demonstrated a statistically significant drop in CD^4+^ levels when Huaier was used in conjunction with drug treatment for PLC, compared to the use of drugs alone [MD = 7.61, 95% CI (5.19, 10.04), *p* < 0.00001] (Figure 7a). Furthermore, Huaier combined with TACE treatment also resulted in a statistically significant decrease in CD^4+^ levels compared to TACE alone [MD = 5.81, 95% CI (4.35, 7.26), *p* < 0.00001] (Figure 7b).

#### 3.3.6. Meta-Analysis of T Lymphocyte Subtype CD^8+^

**Figure 8:**
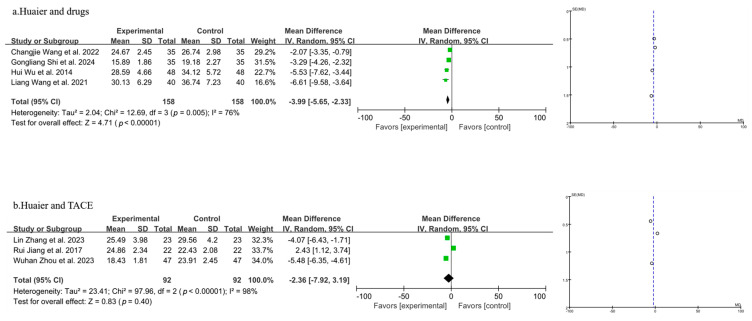
Forest plot and funnel plot of T lymphocyte subtype CD^8+^ levels (the combination of Huaier and drugs in (**a**) and the combination of Huaier and TACE in (**b**)). Wuhan Zhou et al., 2023 [29]; Hui Wu et al., 2014 [31]; Lin Zhang et al., 2023 [32]; Gongliang Shi et al., 2024 [34]; Rui jiang et al., 2017 [35]; Liang Wang et al., 2021 [37]; Changjie Wang et al., 2022 [39].

A total of seven studies were included in the analysis of CD^8+^ levels, as shown in Figure 8 (the combination of Huaier and drugs in 8a and the combination of Huaier and TACE in 8b). According to the random-effects model, the findings indicated that the combination of Huaier with drug treatment for PLC led to a significant elevation in CD^8+^ levels, as opposed to the use of drugs alone [MD = −3.99, 95% CI (−5.65, −2.33), *p* < 0.00001] (Figure 8a). However, Huaier combined with TACE did not result in a statistically significant change in CD^8+^ levels compared with TACE alone [MD = −2.36, 95% CI (−7.92, 3.19), *p* = 0.40] (Figure 8b).

#### 3.3.7. Meta-Analysis of CD^4+^/CD^8+^

**Figure 9:**
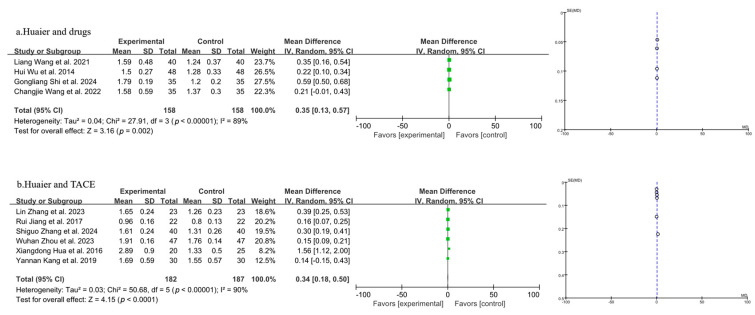
Forest plot and funnel plot of T lymphocyte subtype CD^4+^/CD^8+^ levels (the combination of Huaier and drugs in (**a**) and the combination of Huaier and TACE in (**b**)). Xiangdong Hua et al., 2016 [27]; Wuhan Zhou et al., 2023 [29]; Hui Wu et al., 2014 [31]; Lin Zhang et al., 2023 [32]; Gongliang Shi et al., 2024 [34]; Rui jiang et al., 2017 [35]; Yannan Kang et al., 2019 [36]; Liang Wang et al., 2021 [37]; Changjie Wang et al., 2022 [39]; Shiguo Zhang et al., 2024 [40].

Ten studies were included in the analysis of CD^4+^/CD^8+^ levels, as shown in Figure 9 (the combination of Huaier and drugs in 9a and the combination of Huaier and TACE in 9b). A random-effects model was applied after the heterogeneity test indicated significant variability. A funnel plot was created, and Begg’s and Egger’s tests were performed using Stata 17 to assess publication bias. The *p*-values for both tests indicated no evidence of publication bias in this study. The random-effects model revealed that the combination of Huaier with drug treatment for PLC led to a significant decrease in the CD^4+^/CD^8+^ ratio compared to the use of drugs alone [MD = 0.35, 95% CI (0.13, 0.57), *p* = 0.002] (Figure 9a). Additionally, Huaier combined with TACE treatment resulted in a significant decrease in CD^4+^/CD^8+^ levels compared to TACE treatment alone [MD = 0.34, 95% CI (0.18, 0.50), *p* < 0.0001] (Figure 9b).

#### 3.3.8. Meta-Analysis of AFP

**Figure 10:**
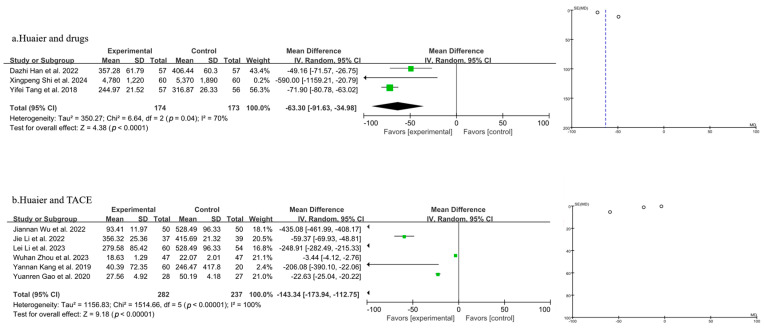
Forest plot and funnel plot of T lymphocyte subtype AFP levels (the combination of Huaier and drugs in (**a**) and the combination of Huaier and TACE in (**b**)). Jie Li et al., 2022 [26]; Wuhan Zhou et al., 2023 [29]; Yannan Kang et al., 2019 [36]; Lei Li et al., 2023 [41]; Jiannan Wu et al., 2022 [42]; Yuanren Gao et al., 2020 [43]; Yifei Tang et al., 2018 [44]; Dazhi Han et al., 2022 [45]; Xingpeng Shi et al., 2024 [46].

Nine studies were included in the analysis of AFP levels, as illustrated in Figure 10 (the combination of Huaier and drugs in 10a and the combination of Huaier and TACE in 10b). A random-effects model was applied after the heterogeneity test revealed significant variability. Using the random-effects model, the results indicated that Huaier combined with drug treatment for PLC led to a statistically significant reduction in AFP levels compared to drugs alone [MD = −63.30, 95% CI (−91.63, −34.98), *p* < 0.0001] (Figure 10a). Additionally, Huaier combined with TACE treatment resulted in a statistically significant decrease in AFP levels compared to TACE treatment alone [MD = −143.34, 95% CI (−173.94, −112.75), *p* < 0.00001] (Figure 10b).

#### 3.3.9. Meta-Analysis of Side Effects

**Figure 11:**
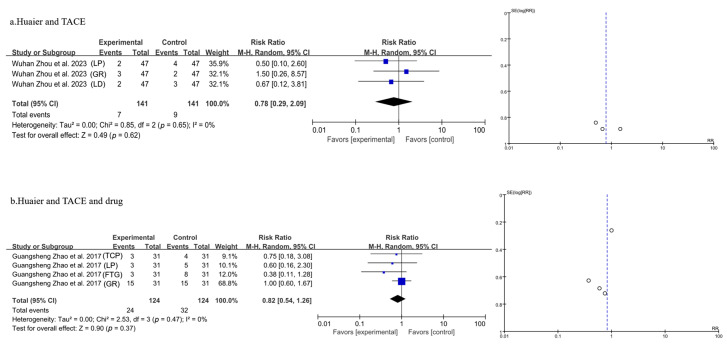
Forest plot of side effects: (**a**) Huaier plus TACE treatment, (**b**) Huaier plus TACE plus drug treatment. Guangsheng Zhao et al., 2017 [21]; Wuhan Zhou et al., 2023 [29].

Three studies involving a total of 282 patients were included in the analysis, as shown in Figure 11a for Huaier plus TACE treatment. The statistical analysis was conducted using a fixed-effects model after the heterogeneity test. The meta-analysis findings indicated that the combination of Huaier with TACE treatment did not result in a significant change in side effects [OR = 0.78, 95% CI (0.29, 2.09), *p* = 0.62] (Figure 10a). Huaier and TACE treatment did not lead to significant changes in leukopenia [LP, OR = 0.50, 95% CI (0.10, 2.60)], gastrointestinal reactions [GR, OR = 1.50, 95% CI (0.26, 8.57)], or liver damage [LD, OR = 0.67, 95% CI (0.12, 3.81)] compared to TACE treatment alone.

Four studies involving a total of 248 patients were included in the analysis, as shown in Figure 11b for Huaier plus TACE plus drug treatment. The statistical analysis was conducted using a fixed-effects model following the heterogeneity test (*p* = 0.47, I^2^ = 0%). The meta-analysis findings indicated that the combination of Huaier, TACE, and drug treatment did not result in a significant change in side effects. The combination of Huaier, TACE, and drug treatment did not significantly affect thrombocytopenia [TCP, OR = 0.75, 95% CI (0.18, 3.08)], leukopenia [LP, OR = 0.60, 95% CI (0.16, 2.30)], fatigue [FTG, OR = 0.38, 95% CI (0.11, 1.28)], or gastrointestinal reactions [GR, OR = 1.00, 95% CI (0.60, 1.67)] compared to TACE plus drug treatment.

## 4. Discussion

### 4.1. Summary of Main Findings

This meta-analysis provided compelling evidence that the combination of Huaier treatment with standard therapies results in significant improvements in several clinical outcomes for primary liver cancer patients.

The analysis above demonstrates that the recurrence rate analysis across 10 studies revealed a significant reduction in recurrence in the Huaier-treated group compared with controls (OR = 0.65, 95% CI: 0.50–0.85, *p* = 0.002). This effect was both robust and significant, suggesting that Huaier may exert a protective role in preventing tumor relapse. The heterogeneity in the results (I^2^ = 80%) suggested variability across studies, potentially due to differences in patient populations or concurrent treatments. Nevertheless, the significant reduction in recurrence rates suggested that Huaier enhances long-term disease control, potentially attributable to its anti-tumor, immunomodulatory, and anti-metastatic properties, which have been documented in previous studies [23,47].

The anti-metastatic effects of Huaier could be particularly important in liver cancer, where tumor spread to other organs remains a major cause of mortality. Treatment with Huaier has been demonstrated in preclinical studies to inhibit tumor cell migration and invasion by modulating several pathways involved in metastasis, including the epithelial–mesenchymal transition (EMT), a process that allows cancer cells to become more mobile and invasive [48]. Huaier’s ability to inhibit EMT and related mechanisms—such as the downregulation of matrix metalloproteinases (MMPs), enzymes that degrade the extracellular matrix and facilitate tumor spread—may help to explain the significant reduction in recurrence observed in our analysis [49]. Furthermore, Huaier’s effects on reducing angiogenesis, the process by which new blood vessels form to support tumor growth, may limit the ability of tumors to metastasize by restricting their blood supply [50].

Overall treatment efficacy was also significantly improved with Huaier, as reflected by a higher total effective rate in 11 studies (OR = 0.79, 95% CI: 0.65–0.96, *p* = 0.02). The significant improvement in treatment efficacy may be attributed to Huaier’s ability to enhance the immune response, reduce tumor angiogenesis, inhibit cancer cell proliferation, and potentially block metastatic pathways. These findings were consistent with the immunomodulatory and anti-metastatic effects of Huaier, particularly its capacity to stimulate immune cell activity and inhibit pathways critical for tumor spread, which may help reduce tumor burden and prevent metastasis [51].

Concerning immune indicators, the results indicated a mixed response. While Huaier did not significantly change the overall CD^3+^ or CD^4+^ levels in the pooled analysis, subgroup analyses showed that Huaier combined with drugs significantly decreased CD^3+^ (*p* = 0.03) and CD^4+^ levels (*p* < 0.00001). These reductions suggested that Huaier may play a role in modulating immune cell subsets [52], potentially reducing overactive immune responses caused by chemotherapy, which could be beneficial for preventing excessive inflammation and immune exhaustion. The significant increase in CD^8+^ levels (MD = −3.99, *p* < 0.00001) supports the role of Huaier in enhancing cytotoxic immune responses. CD^8+^ T cells are crucial for anti-tumor immunity, and their elevation could explain the reduced recurrence rates observed in Huaier-treated patients [53]. The improved CD^4+^/CD^8+^ ratio also underscores the immune-enhancing effects of Huaier, indicating better immune regulation, an essential factor in controlling cancer and preventing its spread [54].

Several mechanisms supported Huaier’s immunomodulatory effects. Preclinical evidence suggests that Huaier polysaccharides enhanced the activity of natural killer (NK) cells and cytotoxic T lymphocytes, both of which play key roles in immune surveillance against tumors [49]. Additionally, Huaier may promote the secretion of immune-stimulating cytokines such as interferon-gamma (IFN-γ), further boosting anti-tumor immunity and inhibiting metastatic processes [48]. These effects align with the improvements in immune markers observed in this meta-analysis, indicating that Huaier may help restore immune function compromised by cancer and its treatment while also potentially preventing metastasis through its immunological actions.

One of the most significant findings of this meta-analysis was the marked reduction in AFP levels (MD = −63.30, *p* < 0.0001) in patients receiving Huaier, particularly when used in combination with chemotherapeutic agents or TACE. Alpha-fetoprotein (AFP) is a critical biomarker for liver cancer progression, and its reduction indicates decreased tumor activity [55]. The mechanism underlying Huaier’s ability to reduce AFP levels is likely associated with its inhibitory effect on hepatocellular carcinoma cell proliferation and its promotion of apoptosis, which directly reduces tumor burden and, consequently, AFP production [56]. The antiproliferative effects of Huaier, possibly through its impact on the PI3K/AKT/mTOR signaling pathway, were well-documented in the literature and provide a plausible explanation for the observed reduction in AFP [57,58]. Additionally, the reduction in AFP levels may correspond with a decreased risk of metastasis, as lower AFP levels have been associated with better prognoses and lower metastatic potential in liver cancer [51].

The significant improvement in quality of life (OR = 0.24, *p* = 0.0007) further highlights Huaier’s therapeutic potential, particularly when combined with chemotherapeutic or biological agents. This enhancement in quality of life may be attributed to Huaier’s ability to reduce tumor burden, inhibit metastatic progression, enhance immune function, and mitigate the side effects of conventional therapies [51]. For example, Huaier’s antioxidant properties and its modulation of inflammatory responses may alleviate symptoms such as fatigue and pain, leading to a better overall quality of life [52]. Additionally, by potentially limiting metastasis, Huaier may help reduce the progression of the disease, contributing to an improved quality of life for patients with advanced liver cancer.

In summary, the safety profile of Huaier was demonstrated, as no significant increase in adverse effects was observed when it was combined with TACE or other treatments. The lack of significant effects on liver damage, gastrointestinal reactions, or leukopenia indicates that Huaier can be safely incorporated into conventional PLC treatment regimens without introducing additional toxicity. Furthermore, its potential anti-metastatic properties make Huaier a valuable adjunct therapy in liver cancer treatment. The evidence from this meta-analysis supports the use of Huaier as an adjunct therapy for PLC, as it significantly improved recurrence rates, enhanced immune function (particularly by increasing CD^8+^ levels), decreased AFP levels, and improved patients’ quality of life. The immunomodulatory effects of Huaier, combined with its ability to lower tumor markers such as AFP and inhibit metastatic spread, provide a strong rationale for its inclusion in multi-modal treatment strategies for liver cancer. These findings underscore Huaier’s potential as a complementary treatment that not only improves clinical outcomes but also maintains a favorable safety profile while potentially reducing the risk of metastasis.

### 4.2. Comparison with Existing Literature

The findings of our study align with the existing literature that supports the use of Huaier as an adjunct therapy for primary liver cancer (PLC). Several prior studies have demonstrated the efficacy of Huaier in inhibiting tumors, improving immune function, and enhancing the overall quality of life for cancer patients. However, the analysis that we performed presented a more comprehensive and up-to-date evaluation, offering additional insights into the variability of Huaier’s effects across different treatment regimens and patient populations. Importantly, the relationship between Huaier and the recurrence and metastasis of PLC was explored, a topic that has been largely underexplored in previous studies. The findings that our group found suggested that Huaier not only played a role in reducing recurrence rates but may also inhibit metastasis, offering a potential mechanism for long-term disease control.

One key strength of previous studies was their detailed focus on specific patient subgroups, such as combining Huaier with specific therapeutic protocols (e.g., chemotherapy, TACE, or immunotherapy). These studies provided valuable insights into how Huaier works under particular conditions, but many were limited by small sample sizes and a lack of randomized controlled trials (RCTs). In contrast, the study our group conducted has several strengths that address these limitations. Firstly, a relatively large number of RCTs were included, most of which are recent, thereby increasing the robustness of our conclusions. The use of rigorous risk bias assessments in our analysis further strengthened the reliability of our findings. Additionally, we conducted comprehensive subgroup and sensitivity analyses, enabling us to investigate potential sources of heterogeneity, such as treatment combinations, and providing a broader perspective on the efficacy of Huaier.

A wide range of outcomes was also considered, including recurrence rates, immune markers (T lymphocyte subtypes CD^3+^, CD^4+^, CD^8+^, and CD^4+^/CD^8+^ ratios), AFP levels, and quality of life. Many previous studies have tended to focus on one or two specific outcomes, limiting the breadth of their conclusions. For instance, earlier studies primarily focused on tumor recurrence or AFP reduction, whereas our analysis provided a more comprehensive evaluation of how Huaier affects both tumor biology and immune function across multiple dimensions.

However, one limitation of our study compared to previous work was the inclusion of a heterogeneous patient population in terms of age, disease stage, and treatment protocols. While earlier studies often focused on more narrowly defined patient groups, such as specific age cohorts or treatment regimens, a broader range of studies to capture a more generalizable effect of Huaier was included. This approach, while increasing the applicability of our findings, also introduced heterogeneity, which may affect the precision of the results. Future research could benefit from more standardized study designs that limit variations in patient characteristics and treatment regimens.

Compared to the existing literature, our study provided several improvements. First, we conducted thorough subgroup analyses based on treatment protocols (e.g., Huaier combined with TACE, drugs, or biological drugs), which was often underexplored in previous studies. These analyses allowed us to identify the most effective combinations of Huaier and standard therapies, offering clearer clinical guidance. Second, we employed both Begg’s and Egger’s tests to evaluate publication bias, a step that is often missing in prior meta-analyses. Our comprehensive assessment of publication bias increased the reliability of our conclusions by addressing one of the key concerns in meta-analytical research.

Ultimately, our study improved upon the existing literature by offering a more holistic and detailed assessment of Huaier’s effects on PLC while also recognizing the importance of patient diversity and treatment variability. While previous research provided valuable insights, our comprehensive methodology, inclusion of recent RCTs, and in-depth subgroup analyses offer a stronger, more generalizable evidence base for the use of Huaier in PLC treatment.

### 4.3. Advantages and Limitations

The main strengths of our study include the following: (1) the use of a rigorous methodology in conducting an updated systematic review and meta-analysis; (2) the inclusion of a relatively large number of randomized controlled trials (RCTs), most of which were recently published; (3) the comprehensive risk of bias assessments conducted across all included RCTs, enhancing the reliability and persuasiveness of the findings; (4) the broad range of reported outcomes considered, providing a comprehensive summary of relevant data to support the efficacy of Huaier in preventing PLC recurrence; and (5) the identification of potential sources of heterogeneity and thorough examination through subgroup and sensitivity analyses.

Several limitations should be considered when interpreting our findings: (1) High heterogeneity was observed in the analyses of the T lymphocyte subtypes CD^3+^, CD^4+^, and CD^4+^/CD^8+^ and the AFP levels. This heterogeneity likely stems from several factors, including the differences in patient characteristics (e.g., age, gender, disease stage), variations in treatment protocols (e.g., Huaier combined with TACE, drugs, or biological drugs), and the complex biological nature of these immune and tumor markers. Such inconsistencies across studies may have impacted the overall reliability of the meta-analysis, and thus, the pooled results should be interpreted with caution. (2) The lack of observational research features: This study did not incorporate key observational research factors, such as patient selection criteria (age, gender, and race), specific exposure factors, treatment planning, and outcome reporting, due to limited available data. These elements were critical for a more comprehensive understanding of Huaier’s therapeutic effects and warrant further investigation. (3) The recent literature shows a clear gender imbalance, with significantly more male than female participants included in PLC-related studies, reflecting the higher incidence of liver cancer in men. However, our analysis did not statistically account for this gender disparity due to incomplete original data. Future research should consider stratifying patients by gender to explore potential differences in response to Huaier therapy, as gender-based variations in liver cancer biology and immune response could influence outcomes. This could provide more nuanced insights into the effectiveness of Huaier in both male and female patients. (4) The duration of treatment varied across the included studies, which may have influenced the consistency of the results. (5) The data on side effects did not show significant changes because there are so few relevant literature reports and cases; thus, more detailed data tracking could be conducted with more detailed data tracking samples. Despite this variation, the results consistently reflected key therapeutic outcomes, suggesting that Huaier’s effects were not limited to a single mechanism, but rather, involved multiple regulatory pathways.

Future research should focus on addressing these limitations by standardizing the study designs, incorporating more detailed patient demographics, and ensuring consistency in treatment protocols to provide a clearer evaluation of Huaier’s efficacy in PLC treatment. Additionally, more detailed reporting of patient demographics, particularly age, gender, and disease stage, is essential for better understanding how Huaier’s efficacy may vary across different patient populations. Moreover, exploring Huaier’s multi-faceted regulatory effects on different immune markers in diverse contexts (e.g., immune activation versus suppression) could provide valuable insights into its comprehensive therapeutic potential. Clinical studies for treating with immune checkpoint inhibitors have shown promising effects for patients with HCC in a phase Ib study (NCT02715531), and tumor factors may influence the TME to restrict T cell infiltration, thereby attenuating tumor response to immune checkpoint blockade [59]. Based on our current clinical data organization, Huaier has shown good clinical performance in enhancing immunity and improving the immune system. Therefore, this result provides a theoretical basis for future immunotherapy research during hepatocellular carcinoma evolution and provides more targeted and personalized therapeutic recommendations for PLC patients.

## 5. Conclusions

In conclusion, our meta-analysis demonstrated that Huaier combination therapy offers excellent therapeutic efficacy and safety for PLC patients. Therefore, Huaier combined therapy holds significant potential as a treatment option alongside conventional treatments and surgical interventions. However, the strength of this conclusion is limited by the quality and nature of the included studies. Future research should focus on large-scale, double-blind, randomized controlled trials (RCTs) to further validate these findings.

## Figures and Tables

**Table 1:** Study characteristics of included studies.

Author, Year	Source	Sample Size		Age		Gender (Male/Female)		Surgery Type		Clinical Intervention		Time of Assessment (Months)	Outcome
		I	C	I	C	I	C	I	C	I	C		
J.Y. Lei et al. 2015 [20]	West China Hospital of Sichuan University	28	25	49.9 ± 8.4	50.3 ± 10.1	(27/1)	(25/0)	/	Liver transplantation	Huaier granule	/	30	①②
Guangsheng Zhao et al. 2017 [21]	Affiliated Zhongshan Hospital of Dalian University	31	31	average age 64.9	average age 62.7	(27/4)	(29/2)	TACE	TACE	Huaier granule	Lobaplatin chemotherapy	12	②⑨
Lin Zhou et al. 2018 [22]	Organ Transplant Institute, Chinese PLA 309th Hospital	18	18	53.94 ± 7.40	49.72 ± 7.00	(17/1)	(15/3)	/	/	Thymalfasin+Huaier granule	Tacrolimus	12	①②③
Zhen Wang et al. 2021 [23]	Chinese PLA General Hospital and Jining First People’s Hospital, Jining, Shandong Province	170	170	30~86	34~86	(143/27)	(129/41)	Thermal ablation	Thermal ablation	TCM	/	12	①②
Shaoju Luo et al. 2023 [24]	The First Affiliated Hospital of Chinese Medicine, Guangzhou University of Chinese Medicine	405	706	49.62 (48.48, 50.74)	50.17 (49.28, 51.05)	(354/51)	(629/77)	Radical surgery	Radical surgery	Huaier granule	/	12	①②
Qian Chen et al. 2018 [25]	Tongji Hospital of Tongji Medical College of HUST	686	316	18~75		(565/121)	(255/61)	Radical surgery	Radical surgery	Huaier granule	/	48	①②
Jie Li et al. 2022 [26]	Tongji Hospital of Tongji	60	54	18~73, average age (50.12 ± 10.71)		(37/23)		Radical surgery	Radical surgery	Huaier granule	/	12	①②⑧
Xiangdong Hua et al. 2016 [27]	Medical College of HUS	20	25	28~72, average age 55		(15/5)	(16/9)	TACE	TACE	Huaier granule	/	12	①②⑦
Jianjun Zhao et al. 2006 [28]	Cancer Hospital, Chinese Academy of Medical Sciences and Chinese Union Medical University, Beijing	44	47	/	/	/	/	Radical surgery	Radical surgery	Huaier granule	/	6	①②
Wuhan Zhou et al. 2023 [29]	Putian First Hospital, Putian, Fujian Province	47	47	37~72, average age 57.98 ± 5.31		(21/26)	(27/20)	Laparoscopic hepatectomy	Laparoscopic hepatectomy	Lenvatinib+Huaier granule	Lenvatinib	6	①②③④⑤⑥⑦⑧⑨
Hairui Wang et al. 2020 [30]	Shengjing Hospital of China Medical University, Shenyang	330	212	18~75		(272/58)	(168/44)	TACE	TACE	Huaier granule	Targeted drug therapy, immunotherapy, radiotherapy, chemotherapy, etc.	54	①②
Hui Wu et al. 2014 [31]	Affiliated Nanjing Hospital, Nanjing Medical University, Nanjing, Jiangsu	48	48	21~68, average age 43.02 ± 7.63		(59/37)		TACE	TACE	Huaier granule	/	3	④⑤⑥⑦
Lin Zhang et al. 2023 [32]	Changshu First People’s Hospital	23	23	48~81, average age 61.83 ± 12.04		(13/10)	(15/8)	TACE	TACE	Huaier granule	/	6	④⑤⑥⑦
Xingang Li et al. 2009 [33]	Beihua University Affiliated Hospital, Jilin Province	19	19	48~75, average age 58.5		(29/9)		TACE	TACE	Huaier granule	/	2	⑤
Gongliang Shi et al. 2024 [34]	Chenzhou First People’s Hospital	35	35	42~68, average age 52.45 ± 3.53		(19/16)	(20/15)	/	/	Drug+Huaier granule	Drug	2	⑤⑥⑦
Rui Jiang et al. 2017 [35]	Huishan District People’s Hospital of Wuxi	22	22	36~72, average age 50.13 ± 9.76		(16/6)	(14/8)	TACE	TACE	Huaier granule	/	0.25	⑤⑥⑦
Yannan Kang et al. 2019 [36]	The First Affiliated Hospital of Zhengzhou University	30	30	60.93 ± 11.00	56.76 ± 8.83	(18/12)	(19/11)	TACE	TACE	Thymosin α1+Huaier granule	Thymosin α1	1	⑤⑦⑧
Liang Wang et al. 2021 [37]	Affiliated Hospital of Shaanxi University of Traditional Chinese Medicine, Xi’an and Yulin No.2 Hospital	40	40	40~71, average age 53.21 ± 8.06		(26/14)	(28/12)	/	/	Sorafenib toluenesulfonate+Huaier granule	Sorafenib toluenesulfonate	2	⑥⑦
Guanlei Wang et al. 2018 [38]	The First Affiliated Hospital of China Medical University, Shenyang	49	47	33~72		(25/24)	(24/23)	/	/	Drug+Huaier granule	Drug	6	⑥⑦
Changjie Wang et al. 2022 [39]	Tangshan Central Hospital	35	35	average age 50.48 ± 6.37		(31/4)	(29/6)	/	/	Drug+Huaier granule	Drug	3	⑥⑦
Shiguo Zhang et al. 2024 [40]	Traditional Chinese Medicine Hospital of Hengyang	40	40	average age 48.32 ± 3.28		(22/18)	(23/17)	TACE	TACE	Thymosin α1+Huaier granule	Thymosin α1	1	⑦
Lei Li et al. 2023 [41]	Tangshan People’s Hospital	38	38	25~75, average age 50.75 ± 4.38		(28/10)	(30/8)	TACE	TACE	Huaier granule	/	1	⑧
Jiannan Wu et al. 2022 [42]	Putuo District People’s Hospital of Zhoushan City	50	50	average age 52.60 ± 6.15		(37/13)	(35/15)	TACE	TACE	Huaier granule	/	3	⑧
Yuanren Gao et al. 2020 [43]	Harbin Medical University Cancer Hospital	28	27	38~72, average age 51.21 ± 11.48		(19/9)	(16/11)	TACE+RFA	TACE+RFA	Huaier granule	/	3	⑧
Yifei Tang et al. 2018 [44]	Shuguang Hospital Affiliated with Shanghai University of Traditional Chinese Medicine	57	56	35~74, average age 52.16 ± 7.33		(40/17)	(41/15)	/	/	Sorafenib Tosylate+Huaier granule	Sorafenib Tosylate	2	⑧
Dazhi Han et al. 2022 [45]	Chaoyang Central Hospital	57	57	41~59, average age 46.57 ± 1.34		(37/20)	(39/18)	/	/	Solafenib+Huaier granule	Solafenib	2	⑧
Xingpeng Shi et al. 2024 [46]	Taizhou Hospital	60	60	average age 58.3 ± 8.1		(36/24)	(31/29)	TACE	TACE	Drug+Huaier granule	Drug	2	⑧

I, intervention group; C, control group; N, none; Y, year; M, month. ① recurrence rate; ② one-year survival rate; ③ quality-of-life analysis; ④ CD^3+^; ⑤ CD^4+^; ⑥ CD^8+^; ⑦ CD^4+^/CD^8+^; ⑧ AFP; ⑨ side effects.

## Data Availability

The raw data supporting the conclusions of this article will be made available by the authors, without undue reservation. The original contributions presented in the study are included in the article/Supplementary Material, and further inquiries can be directed to the corresponding authors.

## Supplementary Materials

The following supporting information can be downloaded at: https://www.mdpi.com/article/10.3390/ph18060884/s1. Table S1: Sensitivity analysis of total recurrence rate (a) and one-year survival rate (b). Table S2: PRISMA 2020 checklist. Reference [60] is cited in the Supplementary Materials.
