# Supplementary material for: The Efficacy of Combined Use of Huaier Granules in the Treatment of Primary Liver Cancer: An Updated Systematic Review and Meta-Analysis
Source: Pharmaceuticals (Basel). 2025 Jun 13;18(6):884. doi: 10.3390/ph18060884 (PMC12196447; doi:10.3390/ph18060884)
Supplement: Supplementary file 1 [file pharmaceuticals-18-00884-s001.zip › Supplementary File 1-Table S1.pdf]

Supporting Information 1:

Table S1 Sensitivity analysis of total recurrence rate(a)and one-year survival rate(b)

Table S1a. Sensitivity analysis of total recurrence rate

Study omitted	Estimate	95% Confidence Interval	
1	0.78160465	0.63547611	0.96133566
2	0.78858721	0.64712465	0.96097362
3	0.77392936	0.62035722	0.96551901
4	0.74690473	0.64506668	0.86482012
5	0.73728937	0.56546825	0.96131939
6	0.80971628	0.67512959	0.9711327
7	0.79487699	0.65625715	0.96277726
8	0.77114356	0.62103164	0.95753956
9	0.80090076	0.66379994	0.96631831
10	0.76392913	0.62862349	0.92835808
Combined	0.77725684	0.63985827	0. 94415939

Table S1b. Sensitivity analysis of one-year survival rate

Study omitted	Estimate	95% Confidence Interval	
1	0.88522917	0.74450332	1.0525551
2	0.85804278	0.71727437	1.0264376
3	0.88775754	0.75096011	1.0494745
4	0.89461613	0.74934441	1.068051
5	0.75881881	0.58010936	0.99258178
6	0.76406109	0.60048008	0.97220439
7	0.9149155	0.7870003	1.0636214
8	0.89470309	0.76088548	1.0520554
9	0.88063002	0.73621821	1.0533687
10	0.90276116	0.77113247	1.0568583
11	0.85991865	0.72514856	1.0197361
Combined	0.87165365	0.73642308	1.0317168
